# Long‐Chain Molecule Reconstruction of Novel Solvation Structure to Stabilize Zinc Metal Anode

**DOI:** 10.1002/advs.76838

**Published:** 2026-07-29

**Authors:** Changdong Chen, Lingwei Xue, Gaojie Li, Xueli Chen, Yongjun Han, Liwei Mi, Yuqing Chen

**Affiliations:** ^1^ School of Materials Science and Engineering Yaoshan Laboratory Pingdingshan University Pingdingshan People's Republic of China; ^2^ Zhejiang Collaborative Innovation Center For Full‐Process Monitoring and Green Governance of Emerging Contaminant Interdisciplinary Research Academy Zhejiang Shuren University Hangzhou People's Republic of China

**Keywords:** anode, aqueous solution, chemical engineering, electrolyte, faraday efficiency, ion transporter, molecule, oxygen transport, solvation, zinc

## Abstract

In aqueous zinc ion batteries, the electrolyte is not only the medium for ion transport, but also directly affects the interface integrity of Zn anode. The active H_2_O molecules in the solvation structure are prone to undergo the hydrogen evolution reaction on the Zn sheet, and induce the evolution of by‐products such as basic zinc salts. Therefore, triethylene glycol diacetate (TGD) with unique long‐chain characteristics and rich oxygen‐containing functional groups was used as an electrolyte additive to reconstruct the solvation structure. TGD can reduce the erosion of H_2_O molecules on the Zn anode, effectively homogenize the electric field distribution, and increase the Coulombic efficiency of the Zn//Cu half‐cell to 95.8%. The Zn//Zn symmetric cell can realize an ultra‐long cycle life of 8000 h. Besides, the capacity retention rate of Zn//I_2_ still reached 91% after 6000 cycles at 1 A g^−1^. This study offers a new method for preparing a high‐durability and reversible Zn anode by regulating the solvation structure through electrolyte additives.

## Graphical abstract

Triethylene glycol diacetate (TGD), with unique long‐chain characteristics and rich oxygen‐containing functional groups, was used as an electrolyte additive to reconstruct the solvation structure. TGD can reduce the erosion of H_2_O molecules on the Zn anode, effectively homogenize the electric field distribution. Therefore, the Zn//Zn symmetric cell can realize an ultra‐long cycle life of 8000 h.

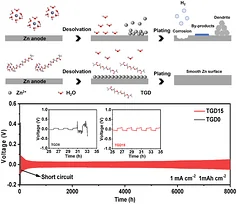

## 1 Introduction

Aqueous zinc‐ion batteries (AZIBs) hold great promise for large‐scale energy storage applications owing to their superior safety, low cost, and eco‐friendly characteristics [1, 2, 3]. Zinc metal anodes are an ideal choice for building high‐energy‐density aqueous batteries owing to their high theoretical capacity and low redox potential and directly available metal resources [4, 5, 6]. However, metallic Zn faces a series of severe challenges in practical applications, which seriously restrict its commercialization process. First, it is difficult to achieve uniform electric field evolution during Zn deposition/dissolution, causing Zn^2+^ ions to preferentially nucleate and grow at the tips, forming uncontrollable dendrites [7, 8, 9]. These dendrites may not only pierce through the separator and cause an internal shorting of the cell, but also detach and form dead Zn during charging and discharging processes, resulting in rapid capacity decay [10, 11]. Second, the Zn anode is intrinsically unstable in near‐neutral or slightly acidic electrolytes, and the hydrogen evolution reaction (HER) is prone to occur on the surface [12, 13, 14]. The accompanying increase in local pH will induce the generation of by‐products, aggravating interface passivation and reducing Coulombic efficiency (CE) [15, 16]. In addition, Zn anodes generally suffer from volume expansion during cycling, further deteriorating the stability of the electrode structure [17, 18].

To address the above‐mentioned challenges, current research efforts are primarily centered on interface engineering, electrolyte optimization, electrode structure design, and separator modification strategies, aiming to suppress dendrite growth, alleviate side reactions, and improve cycle life [19, 20, 21, 22]. Among them, electrolyte modification is widely regarded as one of the most direct and efficient strategies to solve the above problems. The electrolyte is not only the medium for ion transport in AZIBs, but also directly determines the physical and chemical properties of the Zn anode/electrolyte interface [23, 24]. Although the traditional 2 M ZnSO_4_ aqueous electrolyte has low cost and high ionic conductivity, its narrow electrochemical window and abundant active water molecules in the solvation structure make it highly susceptible to HER and the production of by‐products [25, 26, 27]. More importantly, the diffusion energy barrier and interface electric field distribution of Zn^2+^ during the deposition process are uneven, which often leads to the uncontrolled growth of dendrites [28, 29]. Therefore, how to reshape the solvation structure, inhibit the activity of H_2_O molecules, and guide the homogeneous deposition of Zn^2+^ ions via modulating the composition and structure of the electrolyte has become a core scientific problem to improve the reversibility of Zn anodes.

Herein, zinc trifluoromethanesulfonate (ZnOTF) with stronger solvating ability is used as the electrolyte zinc salt, and triethylene glycol diacetate (TGD) is added to the low‐concentration zinc trifluoromethanesulfonate electrolyte (0.5 M ZnOTF). TGD possesses unique long‐chain properties and abundant oxygen‐containing functional groups, which reconstruct the solvation structure in the electrolyte. TGD can also reduce the erosion of H_2_O molecules, effectively improving the problem of inhomogeneous electric field distribution on the Zn anode surface. The solution to the problems of Zn dendrites and side reactions has increased the CE of the Zn//Cu half‐cell to 95.8%, and the Zn//Zn symmetric battery achieves stable operation for 8000 h at 1 mA cm^−2^ and 1 mAh cm^−2^. In addition, the Zn//I_2_ full cell still retains a capacity retention of 91% after 6000 cycles at 1 A g^−1^. Therefore, conducting in‐depth research on electrolyte modification mechanisms is of great significance for breaking through the cycle life bottleneck of AZIBs and promoting their practical application.

## 2 Results and Discussion

TGD, as a surfactant, has abundant polar oxygen‐containing functional groups (─COO─ and ─C─O─C─), is highly soluble in water, and can also dissolve various substances [30]. First, TGD molecules contain multiple ether oxygen bonds and two ester groups, which can effectively replace H_2_O molecules in the solvation sheath and reduce the amount of coordinated H_2_O. Second, multiple oxygen atoms of TGD can serve as hydrogen bond acceptor sites, forming intermolecular hydrogen bonds with H_2_O molecules, effectively breaking the original hydrogen bond network of H_2_O molecules. TGD is used as an additive in 0.5 M ZnOTF electrolyte and to prepare electrolytes with different addition amounts (Figure S1). Fourier transform infrared (FTIR) spectroscopy was used to detect the solvation structure changes of the modified electrolyte and the interaction between ZnOTF and the solvent (Figure 1a). Figure 1b shows that as the amount of TGD added increases, the characteristic peak of the ν(O─H) bond located at 3200–3600 cm^−1^ shifts toward higher wavenumbers, indicating that TGD molecules can act as hydrogen‐bond acceptors to coordinate with H_2_O, reducing the number of hydrogen bonds between H_2_O and thus lowering the activity of free H_2_O in the electrolyte [31]. Owing to the weakened hydrogen‐bonding interactions among H_2_O, TGD displaces some H_2_O in the solvation shell, and the interaction between OTF^−^ anions and Zn^2+^ ions becomes stronger, resulting in the vibration of the ν(SO_3_) bond located at 1020–1040 cm^−1^ moving toward lower wavenumbers (Figure 1c) [32]. From the NMR of Figure S2, it can be seen that the ^1^H peak of the TGD0 electrolyte is located at 4.591 ppm. After adding TGD to the electrolyte, the ^1^H peak shifted. When the addition amount reaches 15 vol%, the ^1^H peak shifts to 4.604 ppm. This indicates that the shielding effect of TGD reduces the electron cloud density around ^1^H from H_2_O, confirming that TGD can weaken the solvation effect between Zn^2+^ and H_2_O. The wettability of the electrolyte on Zn foil affects Zn nucleation and deposition, so the contact angle measurement is used to evaluate it. Taking the electrolyte with a TGD addition of 15 vol% (TGD15) as an example, the contact angle between the TGD15 solution and Zn foil is 57° (Figure 1e), whereas that of the bare TGD0 on Zn foil is 88.3° (Figure 1d). In addition, as the contact time increases, the contact angle of TGD15 gradually decreased (Figure S3b), but TGD0 did not show significant changes (Figure S3a). This indicates that TGD, as a surfactant, can significantly decrease the contact angle between the electrolyte and the Zn sheet. Better wettability means faster ion transport kinetics, which is beneficial to electrochemical performance at high current density [33]. Quantum chemical calculations displayed in Figure 1f reveal that, owing to the distinctive electron‐withdrawing property of carbonyl moieties, the substitution of H_2_O with TGD in the Zn^2+^ solvation shell can effectively reduce the electrostatic repulsion surrounding Zn^2+^ ions. TGD has a high number of donors and can partially replace H_2_O molecules in the Zn^2+^ ion solvation structure. This “dilution” effect weakens the amount of active H_2_O in contact with the Zn anode, suppresses the emergence of parasitic reactions from the source, and improves CE [34]. The three‐electrode test system proves that the Zn foil has better stability in the TGD15 electrolyte. Linear sweep voltammetry (LSV) indicates that compared to TGD0, TGD15 can significantly inhibit the HER of Zn anode (Figure 1g). The Tafel curve proves that the TGD15 electrolyte effectively increases the corrosion voltage from −0.94 to −0.92 V and reduces the corrosion current from 5.49 × 10^−4^ mA cm^−2^ to 1.58 × 10^−4^ mA cm^−2^ (Figure 1h). The chronoamperogram (CA) method further demonstrated the electrochemical stability of the Zn sheet in TGD15 electrolyte. After applying a constant voltage of −0.2 V, the current change on the Zn anode interface is slight and stable, while the current in TGD0 electrolyte decreased significantly (Figure 1i). Compared to the stability of Zn foil in other electrolytes, adding 15vol% TGD is the most suitable (Figure S4). The experimental phenomena and results demonstrate that the formation of new solvation structures in the TGD15 electrolyte can effectively protect the Zn anode while reducing the activity of free H_2_O molecules in the electrolyte, thereby improving the deposition kinetics of Zn^2+^ ions and enhancing the cycling durability of the Zn anode.

**FIGURE 1:**
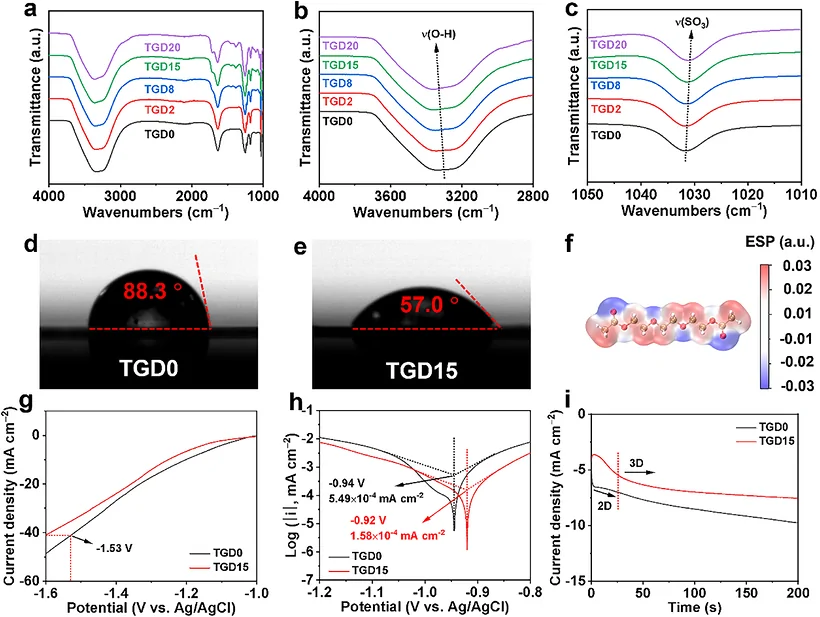
(a–c) FTIR spectra of electrolytes with different concentrations of TGD additives. Contact angle measurement on Zn foil with (d) TGD0 and (e) TGD15 electrolytes. (f) The electrostatic potential (ESP) mapping of the TGD molecule. (g) LSV response curves, (h) Corrosion curves, and (i) CA curves of Zn anode in TGD0 and TGD15 electrolytes.

In order to test the practicality of the electrolyte modified by TGD additives, 2032 coin cells were assembled to test the electrochemical performance. Using Cu foil as the counter electrode, the Zn//Cu half‐cells were assembled to test the CE and cycle stability of the deposition/stripping of Zn^2+^ ions. As displayed in Figure 2a, the CE of the Zn//Cu battery in TGD0 is only 82.5%, while the CE in TGD15 is increased to 95.8%. The CE of Zn//Cu batteries in TGD2, TGD8, and TGD20 are 90.5%, 91.2%, and 92.6%, respectively (Figure S5). In addition, Zn//Cu batteries were cycled at 1 mAh cm^−2^ and 5 mA cm^−2^ (Figure 2b and Figure S6). The Zn//Cu half‐cell can be stably cycled for 6000 cycles in TGD15, but obvious fluctuations occur after 100 cycles in TGD0, implying that the incorporation of TGD greatly enhances the reversibility and stability of the deposition/stripping of Zn^2+^ ions. Even at 5 mA cm^−2^ and 5 mAh cm^−2^, the Zn//Cu battery can cycle stably for 800 cycles (Figure S7). It was further assembled into a symmetric cell for testing of cycle and rate performance. The Zn//Zn symmetric cell exhibits ultra‐stable cycling for 8000 h in TGD15 electrolyte at 1 mA cm^−2^ with 1 mAh cm^−2^, but a short circuit occurs after cycling for 31 h in TGD0 (Figure 2c and Figure S8a). Even at an increased current density of 5 mA cm^−2^, the Zn//Zn cell can still cycle for 3500 h in TGD15 (Figure 2d and Figure S8b). Compared with previously reported electrolyte systems, the Zn//Zn battery with the TGD15 electrolyte reported in this work presents better performance in voltage hysteresis and cycling life (Figure S9 and Table S1). The test results of the Zn//Zn battery with other added amounts of TGD electrolyte showed that the cycle life was improved with the increase of the added amount. When the addition amount reaches 20 vol%, the decrease in lifespan indicates that excessive TGD will affect the reversibility of Zn^2+^ (de)intercalation (Figures S10 and S11). In addition, the Zn//Zn cells cycled into TGD15 maintained good cycling stability even at a large areal capacity of 10 mAh cm^−2^ and a high current density of 10 mA cm^−2^ (Figure S12). For the rate performance, the Zn//Zn cells were tested with 1 mAh cm^−2^. The Zn//Zn cell employing the TGD15 electrolyte exhibits regular and symmetric polarization behavior. With increasing current density, the voltage hysteresis gradually enlarges yet remains stable afterward (Figure 2e). However, the Zn//Zn symmetric battery exhibits significant instability at a current density of 10 mA cm^−2^ in the TGD0 electrolyte, implying that Zn dendrites grow vigorously and deposit unevenly at high current densities in TGD0. It is not difficult to find that the modified electrolyte increases the voltage hysteresis of the battery, mainly because TGD belongs to a long‐chain structure with a larger molecular weight, which can increase the internal resistance of the battery (Figure S13). Furthermore, we also conducted cycling tests on the symmetric cells at 0°C and 40°C. The cycle life of the symmetric cell in TGD15 is better than that in TGD0 (Figure S14), indicating that the TGD15 electrolyte is more stable than the TGD0 electrolyte at both 0°C and 40°C.

**FIGURE 2:**
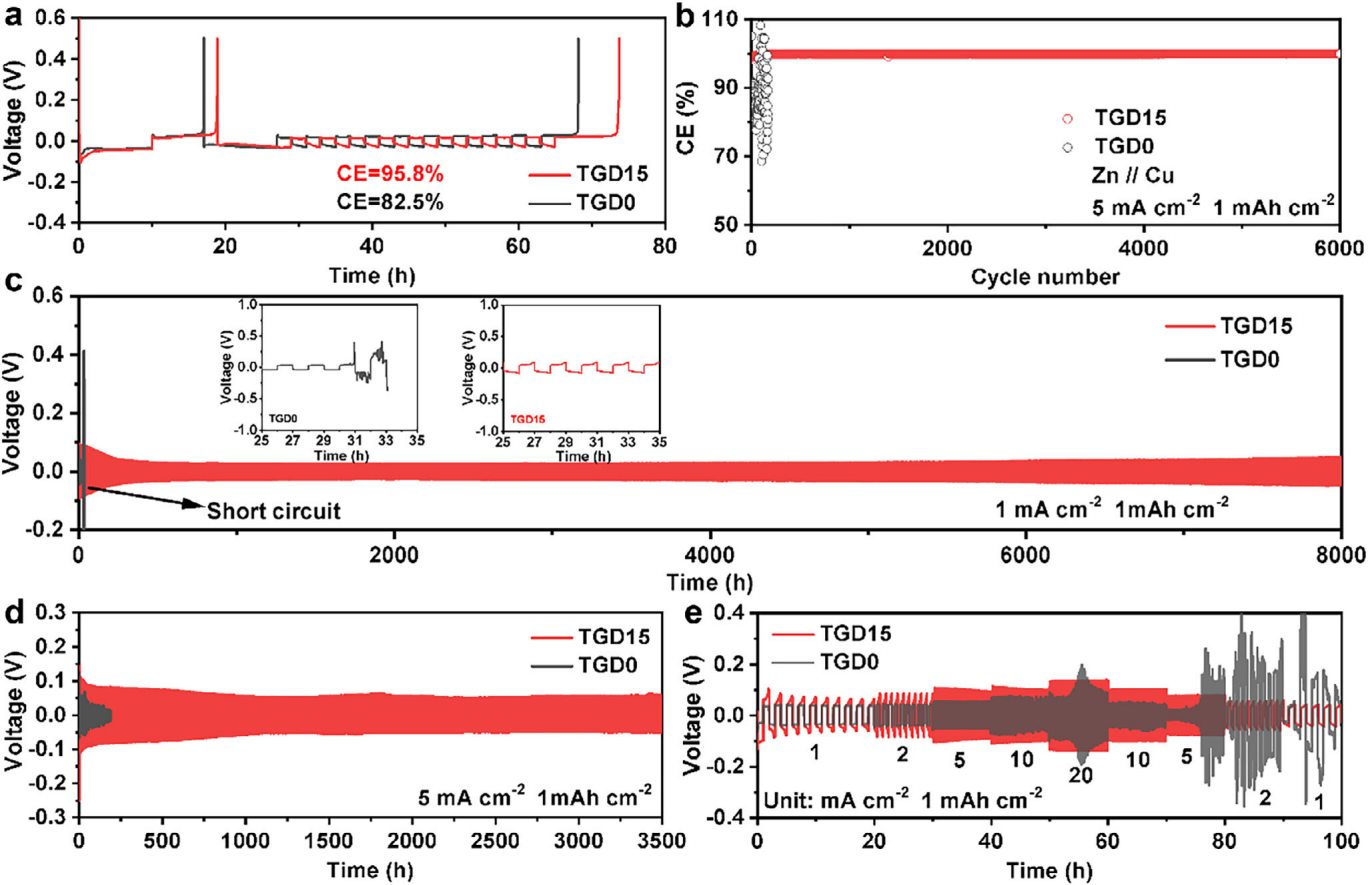
(a) Voltage‐time profile for Zn//Cu half‐cells with TGD0 and TGD15 electrolytes. (b) CE of Zn//Cu half‐cells at 5 mA cm^−2^ and 1 mAh cm^−2^. Voltage‐time profiles of the Zn//Zn symmetrical cells at (c) 1 mA cm^−2^/1 mAh cm^−2^ and (d) 5 mA cm^−2^/1 mAh cm^−2^ with TGD0 and TGD15 electrolytes. (e) Rate performance of the Zn//Zn cells under various current densities.

The intrinsic reasons for the improvement of Zn anode stability by introducing TGD were further analyzed, and the solvation structures of TGD0 and TGD15 electrolytes were constructed through molecular dynamics. The simulated electrolyte structure and the corresponding coordination number (CN) and radial distribution function (RDF) display that there are obvious solvation clusters when Zn^2+^ ions coordinate with H_2_O molecules and TGD molecules. In the TGD0 electrolyte, free H_2_O molecules will form a solvation structure of [Zn(H_2_O)_6_]^2+^ with Zn^2+^ ions (Figure 3c). RDF reveals that the solvation shell distance between Zn^2+^ and H_2_O molecules in the TGD0 electrolyte is 2.05 Å (Figure 3a,b). During the desolvation process of the [Zn(H_2_O)_6_]^2+^ structure at the interface of the Zn anode, free H_2_O molecules are prone to react with the Zn anode and affect its stability. Since there are three oxygen‐containing sites in the TGD molecule, simultaneous simulation analysis of the three sites revealed that the O1 site participates in the solvation structure with a distance of 2.03 Å from the solvation shell of the Zn^2+^ ion, which is greater than that of the O2 and O3 sites (Figure 3d,e). The main reason is that the carbonyl group has a stronger electron‐attracting ability. After the introduction of TGD, the solvation structure of [Zn(H_2_O)_6_]^2+^ is transformed into [Zn(H_2_O)_5_TGD]^2+^ (Figure 3f). The desolvation activation energy derived from variable‐temperature impedance measurements demonstrates that the solvation structure in TGD15 possesses a desolvation energy barrier of 41.4 kJ mol^−1^, significantly lower than the 45.31 kJ mol^−1^ observed in TGD0 (Figure 3g–i and Table S2). This demonstrates that the incorporation of TGD can facilitate the migration of Zn^2+^ ions, optimize their deposited behavior, and suppress the formation of Zn dendrites.

**FIGURE 3:**
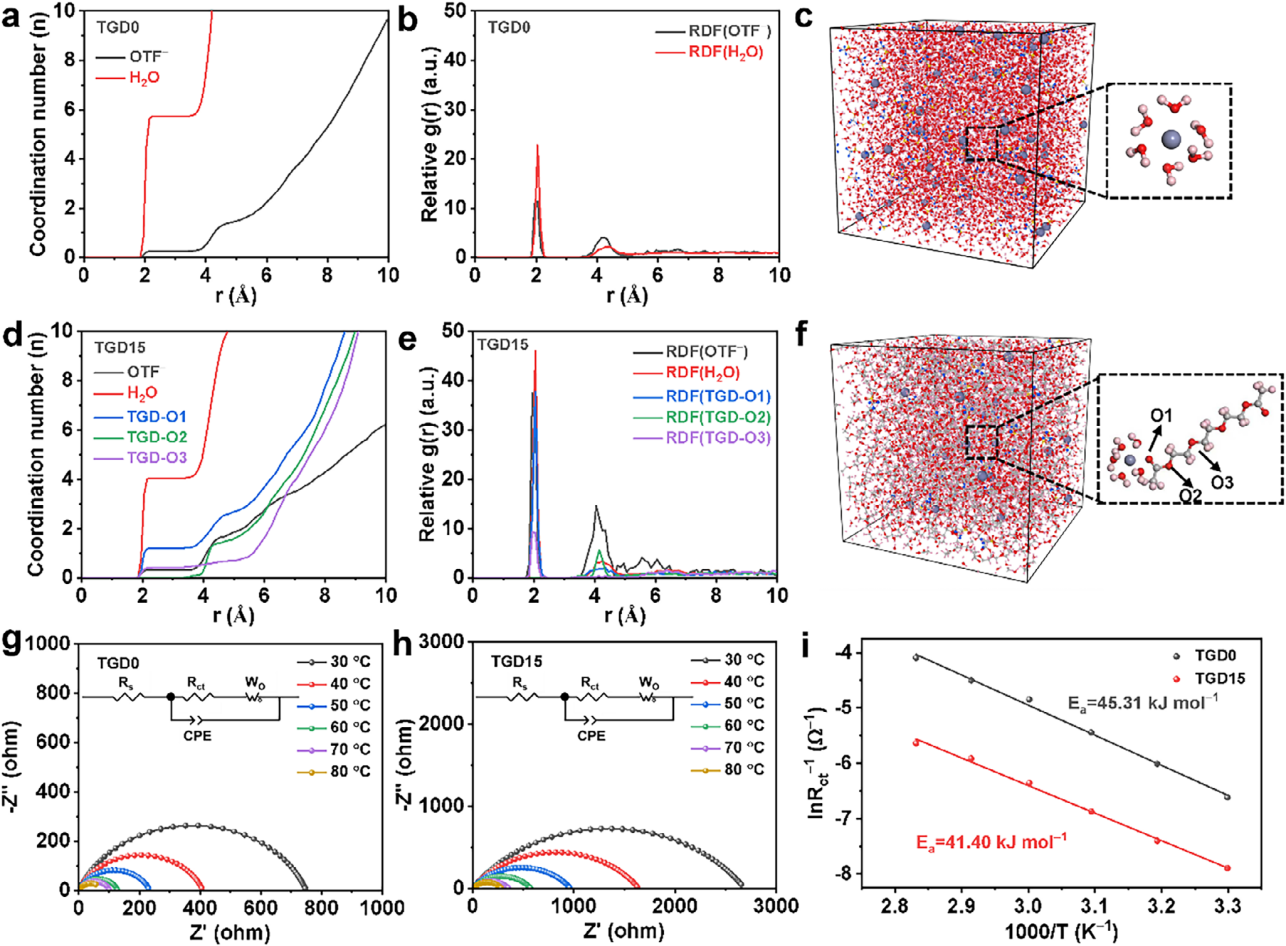
CN and RDFs distribution functions obtained from molecular dynamics (MD) simulations and MD simulation of the solvation structure of (a–c) TGD0 and (d–f) TGD15 electrolyte. Nyquist plots of Zn//Zn cell tests at the temperature range of 30°C–80°C with the (g) TGD0 and (h) TGD15 electrolytes. (i) Corresponding ln(*R*_ct_^−1^) vs. 1000/T plots revealing desolvation activation energy for Zn^2+^.

Observe the changes of Zn foil by soaking it in electrolyte for 7 days. As revealed in Figure S15, a layer of white crystals is formed on the Zn foil surface after being soaked in TGD0 electrolyte. Conversely, the Zn sheet did not show significant changes after being soaked in TGD15 electrolyte. X‐ray diffraction (XRD) analysis revealed that the white crystals were basic zinc salts (Zn_x_OTf_y_(OH)_2x‐y_∙H_2_O) [35], while the Zn foil soaked in TGD15 exhibited no detectable by‐products (Figure 4a). The incorporation of TGD effectively modulates the solvation structure of the [Zn(H_2_O)_6_]^2+^, favoring the homogeneous deposition of Zn^2+^ and reduced parasitic reactions, thereby inhibiting Zn dendrites and reducing by‐products. The cycled Zn anode was characterized to verify the effect of TGD on the deposition behavior of Zn^2+^ ions. The XRD spectra and corresponding characteristic peak of (002)/(100) intensity ratio (R = I_(002)_/I_(100)_) of the Zn anode after 100 cycles in two electrolytes are presented in Figure 4b. The R value after cycling in TGD15 is 0.92, which is much higher than that of the TGD0 electrolyte (0.67), indicating that the new solvation structure can guide the preferential orientation of the (002) crystal facet of Zn. Moreover, ZnO (ICSD PDF#36‐1451) products were also generated on the Zn sheet after cycling in TGD0. X‐ray photoelectron spectroscopy (XPS) characterization further reveals that the Zn sheet surface after cycling in TGD0 electrolyte contains O─H and Zn─O valence bond structures (Figure S16). Observing the Zn anode after cycling through scanning electron microscopy (SEM), it was found that obvious Zn dendrites were generated on the Zn foil surface in TGD0 (Figure 4c). In sharp contrast, the Zn foil cycled in TGD15 exhibited a smooth and compact surface without dendrites (Figure 4d), indicating that the TGD additive has an effective inhibitory effect on the growth of Zn dendrites. Atomic force microscope (AFM) was further used to measure the surface flatness of the Zn anode after cycling in TGD0 and TGD15 electrolytes. As displayed in Figure 4e,f, the Zn after cycling in TGD0 exhibits a relatively rough plane with uneven surface and large fluctuations in roughness. Conversely, the Zn anode in TGD15 exhibits a flat surface morphology with dense and homogeneous Zn^2^^+^ deposition (Figure S17). As shown in Figure 4g, the addition of TGD can successfully change the solvation structure, promote the uniform plating of Zn^2+^, reduce side reactions, and thus inhibit the generation of Zn dendrites and H_2_.

**FIGURE 4:**
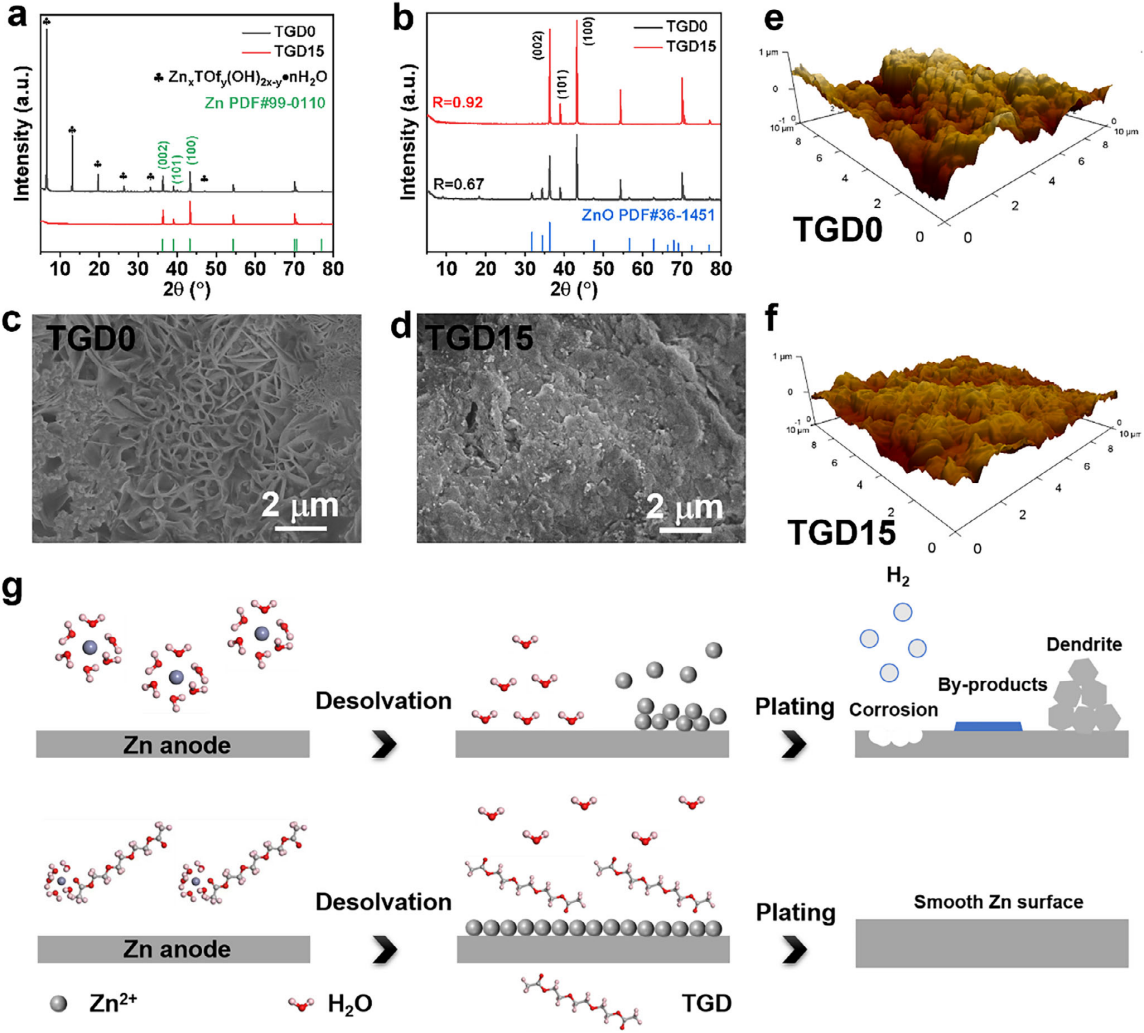
(a) XRD patterns of the soaked Zn anode in TGD0 and TGD15 electrolytes. (b) XRD patterns and (c,d) SEM images of the cycled Zn anode. AFM images of the cycled Zn anodes in (e) TGD0 and (f) TGD15 electrolytes. (g) Schematic illustration of the working mechanism.

In order to directly compare the deposition and stripping processes of Zn, a low‐resolution in situ optical microscope was assembled for observation. As exhibited in Figure 5a, Zn foil was continuously deposited in TGD0 electrolyte for 60 min at a current density of 5 mA cm^−2^. As the deposition time increases, obvious bright spots appear on the surface of the Zn foil, indicating that Zn^2+^ ions are deposited unevenly to form Zn dendrites. In contrast, the Zn foil in TGD15 remained stable during deposition without the formation of bright spots (Figure 5c), demonstrating that the TGD15 electrolyte can significantly inhibit the formation of Zn dendrites. The 3D confocal laser scanning microscopic (CLSM) images further indicate that large aggregates of Zn deposits are exposed on the surface in TGD0 electrolyte with a surface roughness of up to 3 µm (Figure 5b and Figure S18a). On the other hand, the Zn anode interface in TGD15 is relatively flat with a stable surface roughness of 1 µm (Figure 5d and Figure S18b). Furthermore, the evolution of the electric field and ion concentration distribution was further verified via finite element simulation. As displayed in Figure 5e, charge accumulation at Zn protrusion tips in TGD0 leads to a higher local current density [36]. Consequently, massive Zn^2+^ ions rapidly deposit at these protrusion tips, whereas the adjacent flat regions exhibit a much lower concentration of Zn^2+^ ions (Figure 5g) [37]. On the contrary, TGD molecules significantly suppress the electric field at the tip of the protrusion, and the electric field distribution in the non‐tip area is more uniform (Figure 5f). Therefore, the concentration distribution of Zn^2+^ ions in TGD15 electrolyte is more uniform, effectively suppressing the growth of Zn dendrites (Figure 5h). Experiments and theoretical calculations demonstrate that TGD molecules can form strong hydrogen bonding networks with H_2_O molecules and join the solvation sheath layer of Zn^2+^ to reshape the solvation structure. In such a favorable environment, the corrosion and HER of the Zn anode are suppressed, accompanied by greatly reduced by‐products and dendrites, which significantly boosts the electrochemical stability and performance of the Zn anode.

**FIGURE 5:**
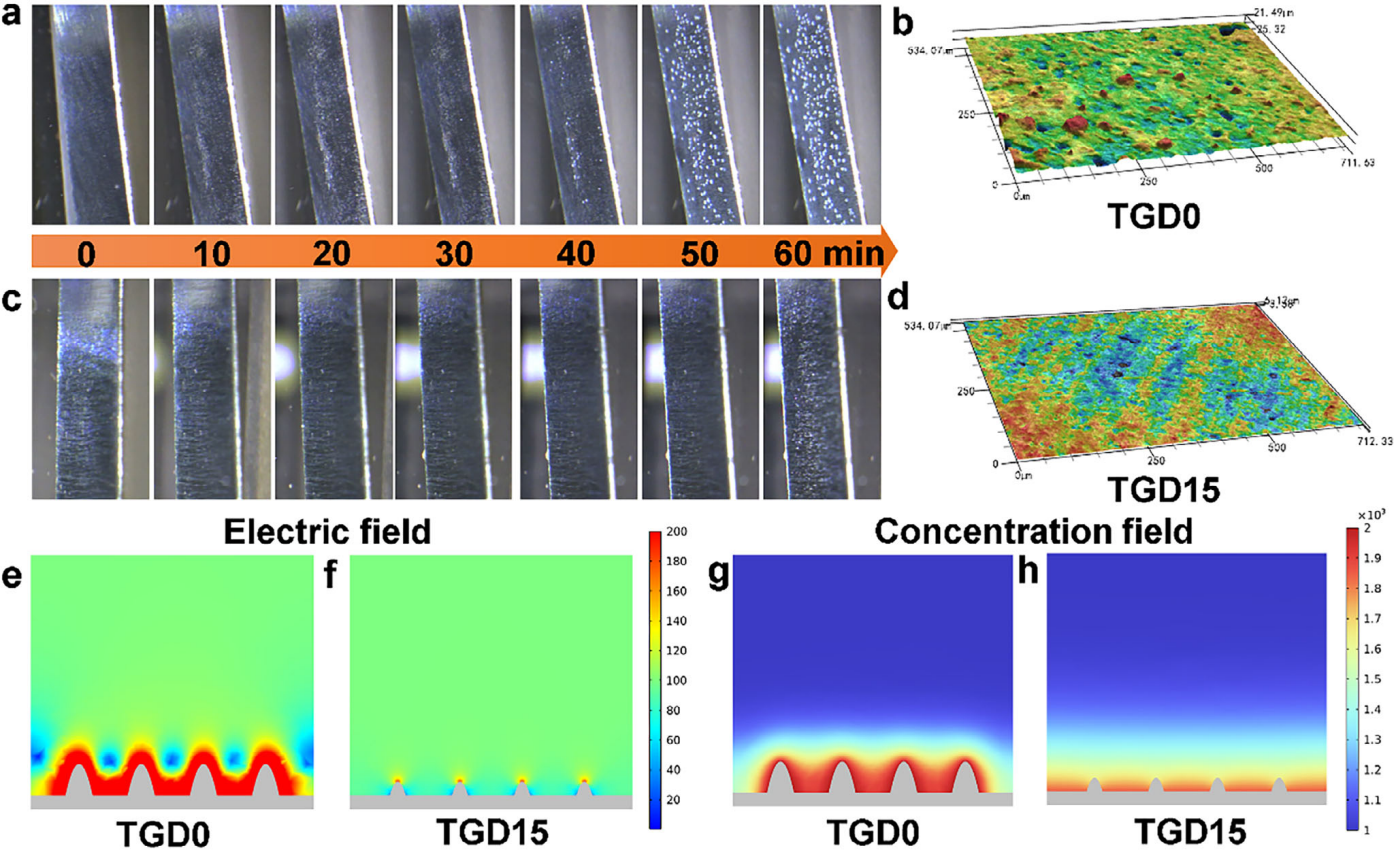
In situ low‐resolution optical microscopy images of Zn plating behaviors, 3D CLSM images of the cycled Zn anodes, Electric field and concentration region simulation in (a,b,e,g) TGD0 and (c,d,f,h) TGD15 electrolytes.

To evaluate the practical feasibility of the TGD15 electrolyte, I_2_ was used as the cathode to assemble the Zn//I_2_ full cell for testing. The cyclic voltammetry (CV) curve of Zn//I_2_ was measured at 0.1 mV s^−1^, and the peak current in TGD15 was greater than TGD0 electrolyte (Figure 6a), indicating faster diffusion of Zn^2+^ ions in the TGD15 electrolyte. By fitting CV curves at different scan speeds, it was calculated that the diffusion coefficient of the battery in TGD15 electrolyte was significantly better than that in TGD0 (Figure S19). The assembled Zn//I_2_ full cells are charged and discharged within a voltage window of 0.6–1.6 V. The Zn//I_2_ full cell can obtain 151 mAh g^−1^ after 6000 cycles in TGD15 at 1 A g^−1^ with a capacity retention of 91% (Figure 6b and Figure S20). However, the Zn//I_2_ full cell was damaged after 2000 cycles in TGD0. Similarly, the Zn//I_2_ battery can stably cycle for 2500 cycles in TGD15 at 5A g^−1^. Moreover, the rate performance of the full battery has also been improved in TGD15 (Figure S21). After cycling to 500 cycles in TGD0, the capacity begins to decay (Figure 6d). Compared with the Zn anodes after cycling in the full cell, obvious Zn dendrites are observed on the Zn anode surface in TGD0 (Figure 6c), but the Zn anode cycled in TGD15 displays a relatively flat surface with no obvious dendrite formation (Figure 6e). The generation of Zn dendrites further affects the internal impedance of the battery. The impedance of the Zn//I_2_ full battery increases significantly after cycling in TGD0 electrolyte, while it is effectively reduced in TGD15 (Figure S22). What's more, increasing the cathode load of I_2_ to 20 mg cm^−2^, high‐load Zn//I_2_ can still cycle stably for 400 cycles at 0.5 A g^−1^ (Figure 6f). The stable operation of the full cell fully demonstrates that TGD is an ideal functional aqueous‐based electrolyte additive.

**FIGURE 6:**
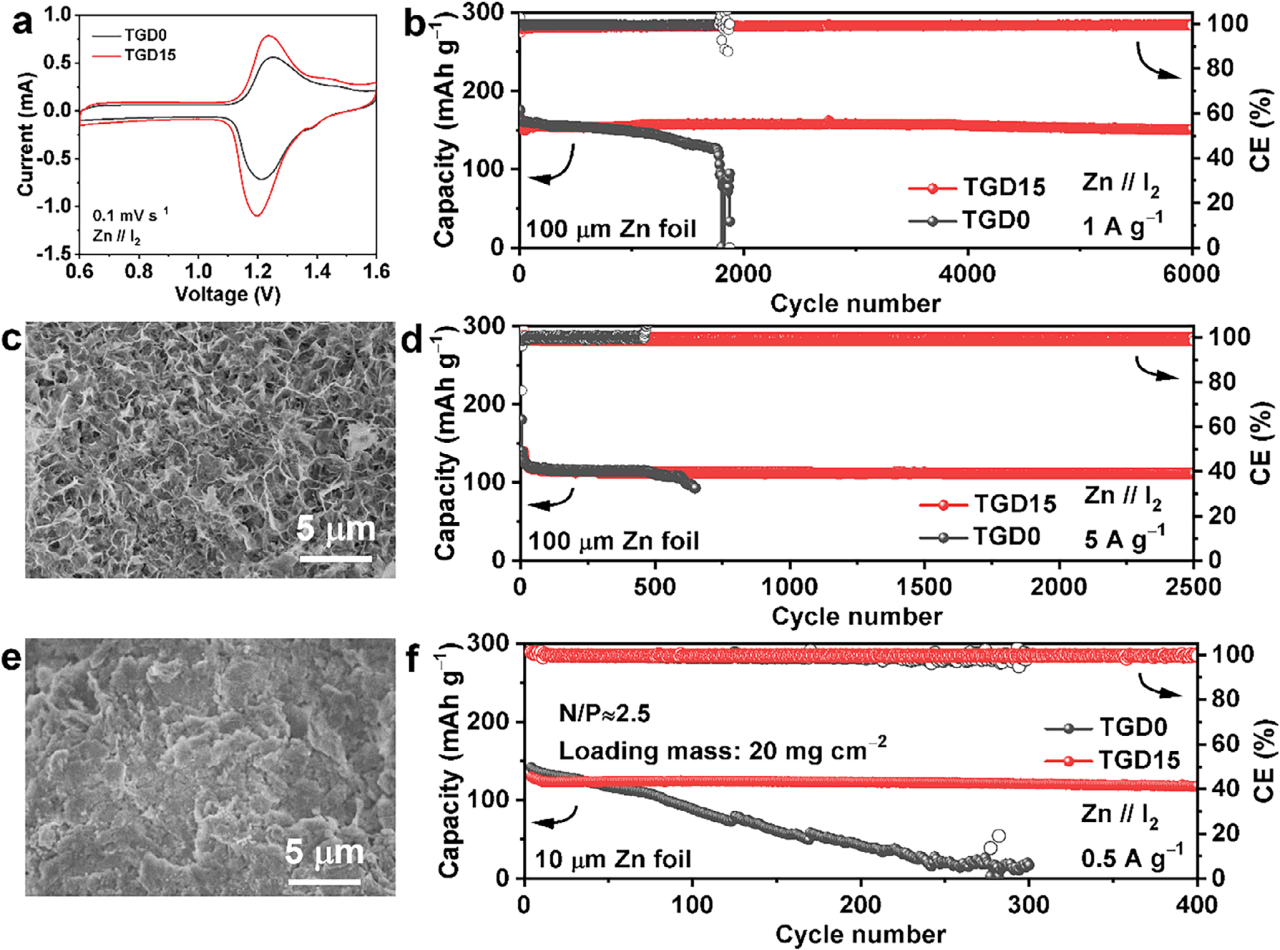
(a) CV profiles of Zn//I_2_ battery. Cycling lifespan of Zn//I_2_ batteries at (b) 1 A g^−1^ and (d) 5 A g^−1^. Top‐view SEM images of cycled Zn anodes from post‐cycling full cells in (c) TGD0 and (e) TGD15 electrolytes. (f) Cycling performances of a Zn//I_2_ full cell with 20 mg cm^−2^ loading mass in TGD15 electrolyte.

## 3 Conclusion

The new TGD‐solvation structure can effectively protect the Zn anode while reducing the activity of free H_2_O in the electrolyte, thereby improving the deposition kinetics of Zn^2+^ ions and optimizing the cycling durability of the Zn anode. Therefore, the Zn//Cu battery not only increases the CE to 95.8% in TGD15, but also can stably cycle for 6000 cycles at 5 mA cm^−2^ with 1 mAh cm^−2^. Furthermore, the Zn//Zn battery has a cycle life of 8000 h at 1 mA cm^−2^ / 1 mAh cm^−2^, and maintains good cycle stability under larger areal capacity and higher current density. The high‐load Zn//I_2_ battery also exhibits good electrochemical performance in TGD15. The addition of TGD provides new ideas for the development of AZIBs.

## Author Contributions

Y.Q.C conceived the project and wrote the manuscript. C.D.C., L.W.X., and G.J.L. carried out most of the experiments and conducted all the characterization. X.L.C., Y.J.H and L.W.M. investigated the project. All authors discussed and commented on the manuscript.

## Funding

The authors gratefully acknowledge the financial support from the Science and Technology Development Program of Henan Province (262102241050), the Natural Science Foundation of Henan Province (252300420256 and 262300420026), and the Natural Science Foundation of Zhejiang Province (LQN26B030018), High‐Level Talents Fund of Pingdingshan University（PXY‐BSQD‐2026039).

## Conflicts of Interest

The authors declare no conflicts of interest.

## Supporting information




**Supporting File**: advs76838‐sup‐0001‐SuppMat.docx.

## Data Availability

The data that support the findings of this study are available from the corresponding author upon reasonable request.
