# Supplementary material for: Long‐Chain Molecule Reconstruction of Novel Solvation Structure to Stabilize Zinc Metal Anode
Source: Adv Sci (Weinh). 2026 Jul 29;13(59):e76838. doi: 10.1002/advs.76838 (PMC13418497; doi:10.1002/advs.76838)
Supplement: Supplementary file 1 [file ADVS-13-e76838-s001.docx]

**Long chain molecule reconstruction of novel solvation structure to stabilize zinc metal anode**

Changdong Chen^a#^, Lingwei Xue^a#^, Gaojie Li^a#^, Xueli Chen^a^, Yongjun Han^a^, Liwei Mi^a^, Yuqing Chen^b[1]*^

a School of Materials Science and Engineering, Yaoshan Laboratory, Pingdingshan University, Pingdingshan 467000, P. R. China

b Zhejiang Collaborative Innovation Center for Full-Process Monitoring and Green Governance of Emerging Contaminant, Interdisciplinary Research Academy, Zhejiang Shuren University, Hangzhou 310021, China

**Experimental section**

**Preparation of Electrolytes**

Aqueous base electrolyte was prepared by dissolving zinc trifluoromethanesulfonate (Zn(CF_3_SO_3_)_2_, ZnOTF, Aladdin, 98%) in deionized water under continuous magnetic stirring until fully dissolved. Then, the additive triethylene glycol diacetate (TGD, Aladdin, 98%) was introduced into the base electrolyte with volume fractions of 0vol%, 2 vol%, 8 vol%, 15 vol% and 20 vol% (denoted as TGD0, TGD2, TGD8, TGD15 and TGD20), respectively. The obtained mixtures were magnetically stirred at room temperature for 2 h to achieve uniform blending, resulting in a series of stable ZnOTF-based electrolytes with different TGD contents.

**Material Characterizations**

Contact angles of the Zn anode in electrolytes were measured using a DSA25 goniometer (Kruss, Germany). Fourier transform infrared (FTIR) spectra were recorded on an iS50 spectrometer (Thermo Scientific, USA). The crystal structure of the Zn anode was determined by X-ray diffraction (XRD, Bruker D8, Cu Kα radiation). Raman spectra were collected with a LabRAM HR Evolution spectrophotometer (Horiba, France). The microstructure and surface morphology of the Zn anode were observed by field-emission scanning electron microscopy (FESEM, Hitachi SU8010, Japan). The surface roughness of cycled Zn anodes was characterized by atomic force microscopy (AFM, NTEGRA Spectra, NT-MDT, Russia) and confocal laser scanning microscopy (CLSM, Olympus OLS4100, Japan). The chemical states and surface composition of the Zn anode were analyzed by X-ray photoelectron spectroscopy (XPS, Escalab Xi+, Thermo Scientific, USA).

**Electrochemical Measurements**

For Zn-I_2_ full cells, iodine powder and activated carbon were uniformly mixed at a mass ratio of 1:1 to serve as active materials. The active materials (80 wt%), carboxymethyl cellulose (CMC, 10 wt%), and acetylene black (10 wt%) were blended with deionized water to form a homogeneous slurry, which was subsequently coated onto carbon paper to fabricate the cathode. The areal mass loading of the I_2_ cathode was controlled at about 1-2 mg cm⁻^2^. Galvanostatic charge-discharge (GCD) measurements were carried out on a LAND CT2001 battery test system at 27 °C under various current densities within a voltage window of 0.6–1.6 V. Electrochemical impedance spectroscopy (EIS) and cyclic voltammetry (CV) tests were conducted on a CHI660E electrochemical workstation.

The coefficient diffusion of Zn^2+^ ions measured from CV is calculated as follow:

$$I_{p}=\left( 2.69\times{10}^{5} \right)n^{3/2}AD^{1/2}Cv^{1/2}$$

Where $I_{p}$ is the peak current (A), $n$ is the number of reaction electrons, $A$ is the contact area between the electrolyte and electrode (cm^2^), $D$ is the diffusion coefficient Zn^2+^ ions (cm^2^ s^−1^), $C$ is the concentration of Zn^2+^ ions in the electrode (mol cm^−3^), and $v$ is the scan rate (V s^−1^).

**Simulation Methods**

Molecular dynamics (MD) simulations were performed using the Large Scale Atomic/Molecular Massively Parallel Simulator (LAMMPS) code and based on Optimized Potential for Liquid Simulations-all atom (OPLS-AA) force field. The organic solvent forcefield parameters were generated by the LigParGen web server except for adopting restrained electrostatic potential (RESP) atomic partial charges, which were obtained based on electrostatic potential (ESP) charges using the Multiwfn program. The initial atomic coordinates were generated with Packing Optimization for Molecular Dynamics Simulations (Packmol) program, and the final model boxes, and solvation structures were visualized by Visual Molecular Dynamics (VMD) software. The periodic boundary conditions were applied in all three directions for all simulations. A cutoff of 12 Å was used for both van der Waals interactions and long-range correction (particle-particle particle-mesh) of Coulombic interactions. The time step was fixed to be 1 fs. All electrolyte models were first equilibrated in an NVT ensemble using the Nose–Hoover barostat for 10 ns to maintain a temperature of 298 K. After that, the electrolytes were maintained at 298 K (1 atm) for 20 ns NPT to make the system equilibrium under a Nosé-Hoover thermostat and barostat. A 10 ns production run (the calculations data for the RDF were all taken from this stage) was finally conducted in the NPT ensemble under a Nosé-Hoover thermostat and barostat at 298 K (1 atm). Before simulation, Packmol was used only to generate the initial model structure. We used Packmol to randomly distribute Zn^2+^, OTF^-^, H_2_O molecules, and TGD molecules within the box, setting the tolerance to 2. Moreover, the Multiwfn program was used to calculate RESP, which is a commonly used program for processing DFT simulation results.

The finite element analysis simulations are performed with the COMSOL Multiphysics software. The FEA model in shown below was defined by the law of mass conservation and electroneutrality assumption of related ions without considering the possible side reactions. Simulation considers the influence of two different factors on ion concentration: concentration diffusion and electric field migration. A simulation model was built for a local model of 9*10 μm, with an initial layer of 0.5 μm.

Using the current distribution and concentration diffusion equation to track the current and concentration distribution, the flux of each ion in the electrolyte can be calculated by Nernst-Planck equation,

$$N_{i}=-D_{i}\left( \nabla c_{i}+\frac{F}{RT}z_{i}c_{i}\nabla\emptyset_{l} \right),i=1,2,\ldots,n$$

Where D_i_, N_i_, c_i_, z_i_, F, T, R, and ϕ_l_ denote the diffusion coefficient, the flux, the concentration, the charge number, the Faradaic constant, temperature, gas constant, and electrolyte potential, respectively. I stands for the position along the diffusion region of thickness d (0 < l < d). Based on the steady-state continuity equations and the law of mass conservation, we have

$$\frac{\partial c_{i}}{\partial t}+\nabla\times N_{i}=0$$

And the electroneutrality assumption was represented as

$$F\sum_{i=1}^{n} z_{i}c_{i}=0$$

The boundary condition was set as followed:

$$\vec{n}\times J=0$$

The simulation steps include a current distribution initialization and a steady step. The initial ion concentration is set to 2000 mol m^-3^. The electrolyte conductivity is 0.1 S m^-1^, and the boundary potential is 0.25 V. The equations required by the model have been fully described.

**Figure S1.** Digital photograph of ZnOTF electrolytes with different amount of TGD additive.

**Figure S2.** ^1^H NMR spectra of H_2_O and different electrolytes.

**Figure S3.** The changing trend of contact angle between Zn foil with (a) TGD0 and (b) TGD15 electrolytes over time.

**Figure S4.** (a) LSV response curves, (b) Corrosion curves and (c) CA curves of Zn anode in TGD2, TGD8 and TGD20 electrolytes.

**Figure S5.** Voltage-time profile for Zn//Cu half-cells with TGD2, TGD8 and TGD20 electrolytes.

**Figure S6.** Voltage vs. Capacity curves of Zn//Cu cell with TGD15 electrolyte at 5 mA cm^-2^ and 1 mAh cm^-2^.

**Figure S7.** CE of Zn//Cu cells with TGD15 electrolyte at 5 mA cm^−2^ and 5 mAh cm^−2^, and the corresponding voltage vs. capacity curves.

**Figure S8.** Voltage–time profiles at the current density of (a) 1 mA cm^−2^ and (b) 5 mA cm^−2^ with an areal capacity of 1 mAh cm^−2^ in TGD15 electrolyte.

**Figure S9.** Comparison of voltage hysteresis versus cycling life for symmetric Zn//Zn cells at 1 mA cm^-2^ / 1 mAh cm^-2^ with TGD15 electrolyte and recently reported electrolytes.

**Figure S10.** Voltage–time profiles at the current density of 1 mA cm^−2^ with an areal capacity of 1 mAh cm^−2^ in various electrolytes.

**Figure S11.** Voltage–time profiles at the current density of 5 mA cm^−2^ with an areal capacity of 1 mAh cm^−2^ in various electrolytes.

**Figure S12.** Voltage–time profiles at 10 mA cm^−2^ with (a) 1 mAh cm^−2^ and (b) 10 mAh cm^−2^ in TGD15 electrolyte.

**Figure S13.** EIS curves of Zn//Zn cells at various electrolytes.

**Figure S14.** Voltage–time profiles at the current density of 1 mA cm^−2^ with an areal capacity of 1 mAh cm^−2^ in TGD0 and TGD15 electrolytes at 0 °C and 40 °C.

**Figure S15.** Digital photographs of the soaked Zn anode in TGD0 and TGD15 electrolytes.

**Figure S16.** XPS analysis of cycled Zn anodes surface in (a) TGD0 and (b) TGD15 electrolytes.

**Figure S17.** Surface roughness curve corresponding to AFM.

**Figure S18.** Surface roughness curve corresponding to3D CLSM.

**Figure S19.** The relationship between the peak current (Ip) and the square root over scan rate (ν^1/2^) of I_2_//Zn full cells.

**Figure S20.** Galvanostatic charge/discharge curves of Zn//I_2_ full cells at 1 A g^-1^ with TGD0 and TGD15 electrolytes.

**Figure S21.** Rate performance of Zn//I_2_ full cells with TGD0 and TGD15 electrolytes.

**Figure S22.** EIS curves of before and after cycled Zn//I_2_ full cells at TGD0 and TGD15 electrolytes.

**Table S1**. Comparison of the electrochemical performance Zn//Zn battery with TGD15 electrolyte and the modified aqueous electrolytes as reported elsewhere at 1 mA cm^-2^/1 mAh cm^-2^.

| Electrolyte composition | Voltage hysteresis | Cycling life | Ref. |
| --- | --- | --- | --- |
| 1 M Zn(OTf)_2_/H_2_O-DOL | 50 mV | 980 h | S1 |
| 1 M ZnSO_4_/H_2_O-Glucose | 80 mV | 2000 h | S2 |
| 0.5 M Zn(OTf)_2_/H_2_O-TEP | 80 mV | 1500 h | S3 |
| 2 M ZnSO_4_/H_2_O-DMF | 100 mV | 2500 h | S4 |
| 2 M ZnSO_4_/H_2_O-DX | 110 mV | 1200 h | S5 |
| 2 M ZnSO_4_/H_2_O-DMSO | 64 mV | 2100 h | S6 |
| 1 M Zn(OTf)_2_/H_2_O-PEGDME | 200 mV | 2000 h | S7 |
| 0.5 M Zn(OTf)_2_/H_2_O-THF | 38 mV | 2800 h | S8 |
| 2 M ZnSO_4_/H_2_O-TW20 | 40 mV | 2500 h | S9 |
| 1 M ZnSO_4_/H_2_O-Nin | 90 mV | 4500 h | S10 |
| 2 M ZnSO_4_/H_2_O-BHEG | 50 mV | 4500 h | S11 |
| 2 M ZnSO_4_/H_2_O-MAA | 50 mV | 4000 h | S12 |
| 2 M ZnSO_4_/H_2_O-SAA | 50 mV | 3400 h | S13 |
| 2 M ZnSO_4_/H_2_O-BT | 60 mV | 2700 h | S14 |
| 1 M ZnSO_4_/H_2_O-SL | 50 mV | 2800 h | S15 |
| 2 M ZnSO_4_/H_2_O-GL+MSA | 60 mV | 4000 h | S16 |
| 0.5 M Zn(OTf)_2_/H_2_O-TGD | **48 mV** | **8000 h** | **This work** |

**Notes:** 1,3-dioxolane (DOL), Triethyl phosphate (TEP), N,N-dimethylformamide (DMF), 1,4-dioxane (DX), Dimethyl sulfoxide (DMSO), Poly(ethylene glycol) dimethyl ether (PEGDME), Tetrahydrofuran (THF), Tween-20 (TW20), Ninhydrin (Nin), N,N-bis(2-hydroxyethyl)glycine (BHEG), Methacrylic acid (MAA), Saponin and anisaldehyde (SAA), Bis(2-hydroxyethyl)amino-tris(hydroxymethyl)methane (BT), Sophorolipid (SL), Glycerol (GL), Methylsulfonamide (MSA).

**Table S2.** The EIS values of Zn//Zn cells at the temperature range of 30 to 80 °C with the TGD0 and TGD15 electrolytes.

|  | 30 °C | 40 °C | 50 °C | 60 °C | 70 °C | 80 °C |
| --- | --- | --- | --- | --- | --- | --- |
| TGD0 | 60.0 Ω | 90.0 Ω | 127.4 Ω | 232.2 Ω | 407.8 Ω | 748.0 Ω |
| TGD15 | 281.9 Ω | 370.0 Ω | 576.0 Ω | 966.2 Ω | 1642 Ω | 2688 Ω |

**References**

[S1]	H. Du, K. Wang, T. Sun, J. Shi, X. Zhou, W. Cai, Z. Tao, Improving zinc anode reversibility by hydrogen bond in hybrid aqueous electrolyte. *Chem. Eng. J.* **2022**, *427*, 131705.

[S2]	P. Sun, L. Ma, W. Zhou, M. Qiu, Z. Wang, D. Chao, W. Mai, Simultaneous Regulation on Solvation Shell and Electrode Interface for Dendrite-Free Zn Ion Batteries Achieved by a Low-Cost Glucose Additive. *Angew. Chem., Int. Ed.* **2021**, *60*, 18247.

[S3]	S. Liu, J. Mao, W. K. Pang, J. Vongsvivut, X. Zeng, L. Thomsen, Y. Wang, J. Liu, D. Li, Z. Guo, Tuning the Electrolyte Solvation Structure to Suppress Cathode Dissolution, Water Reactivity, and Zn Dendrite Growth in Zinc-Ion Batteries. *Adv. Funct. Mater.* **2021**, *31*, 2104281.

[S4]	P. Xiong, Y. Kang, N. Yao, X. Chen, H. Mao, W.-S. Jang, D. M. Halat, Z.-H. Fu, M.-H. Jung, H. Y. Jeong, Y.-M. Kim, J. A. Reimer, Q. Zhang, H. S. Park, Zn-Ion Transporting, In Situ Formed Robust Solid Electrolyte Interphase for Stable Zinc Metal Anodes over a Wide Temperature Range. *ACS Energy Lett.* **2023**, *8*, 1613.

[S5]	T. Wei, Y. Ren, Y. Wang, L. e. Mo, Z. Li, H. Zhang, L. Hu, G. Cao, Addition of Dioxane in Electrolyte Promotes (002)-Textured Zinc Growth and Suppressed Side Reactions in Zinc-Ion Batteries. *ACS Nano* **2023**, *17*, 3765.

[S6]	D. Feng, F. Cao, L. Hou, T. Li, Y. Jiao, P. Wu, Immunizing Aqueous Zn Batteries against Dendrite Formation and Side Reactions at Various Temperatures via Electrolyte Additives. *Small* **2021**, *17*, 2103195.

[S7]	Z. Hou, Z. Lu, Q. Chen, B. Zhang, Realizing wide-temperature Zn metal anodes through concurrent interface stability regulation and solvation structure modulation. *Energy Storage Mater.* **2021**, *42*, 517.

[S8]	S. You, Q. Deng, Z. Wang, Y. Chu, Y. Xu, J. Lu, C. Yang, Achieving Highly Stable Zn Metal Anodes at Low Temperature via Regulating Electrolyte Solvation Structure. *Adv. Mater.* **2024**, *36*, 2402245.

[S9]	Q. Deng, S. You, W. Min, Y. Xu, W. Lin, J. Lu, C. Yang, Polymer Molecules Adsorption-Induced Zincophilic-Hydrophobic Protective Layer Enables Highly Stable Zn Metal Anodes. *Adv. Mater.* **2024**, *36*, 2312924.

[S10]	Z. Zhang, X. Lan, G. Liao, W. Du, Y. Zhang, M. Ye, Z. Wen, Y. Tang, X. Liu, C. C. Li, Coupling Zn2+ Ferrying Effect With Anion–π Interaction to Mitigate Space Charge Layer Enables Ultra-High Utilization Rate Zn Anode. *Angew. Chem., Int. Ed.* **2025**, *64*, e202503396.

[S11]	J. Bu, P. Liu, G. Ou, M. Ye, Z. Wen, Y. Zhang, Y. Tang, X. Liu, C. C. Li, Interfacial Adsorption Layers Based on Amino Acid Analogues to Enable Dual Stabilization toward Long-Life Aqueous Zinc Iodine Batteries. *Adv. Mater.* **2025**, *37*, 2420221.

[S12]	N. Yu, S. Lin, S. Zhou, Y. Li, J. Li, Q. Zeng, L. Chen, L. Wang, K. Guo, X. Wang, Y. Li, Engineering aqueous electrolytes with a trifunctional additive for robust zinc anodes across a wide temperature range. *Energy Storage Mater.* **2025**, *80*, 104398.

[S13]	K. Liu, M. Sun, Y. Wu, T. Zhang, A. Zhu, S. Bu, C. Luan, K. Liu, Y. Zhou, D. Lin, S. Wu, C. S. Lee, B. Huang, G. Hong, W. Zhang, Binary Electrolyte Additive-Reinforced Interfacial Molecule Adsorption Layer for Ultra-Stable Zinc Metal Anodes. *Adv. Mater.* **2025**, *37*, 2420079.

[S14]	Y.-M. Li, W.-H. Li, X.-Y. Zhang, Y.-Z. Tang, Z.-M. Liu, J.-P. Zhang, X.-L. Wu, Multifunctional pH-Controlling Electrolyte Enables Ultrastable and Highly Reversible Zinc Anode. *Adv. Funct. Mater.* **2025**, *35*, 2420446.

[S15]	Z. Shi, S. Chen, M. Zan, L. Zhang, J. Gong, Y. Zhou, K. Müllen, F. Lai, T. Liu, Surfactant-mediated mesoscopic confinement and selective interfacial shielding for highly stable zinc anode. *Energy Environ. Sci.* **2026**, *19*, 1385.

[S16]	X. Wei, J. Guan, Y. Mu, Y. Zou, X. Wei, L. Yang, Q. Man, C. Yang, L. Zang, J. Sun, L. Zeng, Decoding Hydrogen-Bond Network of Electrolyte for Cryogenic Durable Aqueous Zinc-Ion Batteries. *Nano-Micro Lett.* **2026**, *18*, 127.

1. ^#^These authors contributed equally to this study.
